# A quality of life questionnaire for adolescents with cerebral palsy: psychometric properties of the Bengali CPQoL-teens

**DOI:** 10.1186/s12955-019-1206-x

**Published:** 2019-08-02

**Authors:** Rosalie Power, Rahena Akhter, Mohammad Muhit, Sabrina Wadud, Eamin Heanoy, Tasneem Karim, Nadia Badawi, Gulam Khandaker

**Affiliations:** 1 Discipline of Child and Adolescent Health, Faculty of Medicine and Health, University of Sydney, Sydney, NSW Australia; 2 Asian Institute of Disability and Development, University of South Asia, Dhaka, Bangladesh; 3 School of Dentistry, Faculty of Medicine and Health, University of Sydney, Sydney, NSW Australia; 4 CSF Global, Dhaka, Bangladesh; 5 Cerebral Palsy Alliance Research Institute, University of Sydney, Sydney, NSW Australia; 6 Central Queensland Public Health Unit, Central Queensland Hospital and Health Service, QLD, Rockhampton, Australia; 7 The Children’s Hospital at Westmead Clinical School, Cnr Hawkesbury Rd and Hainsworth St, Locked Bag 4001, Westmead, NSW 2145 Australia

**Keywords:** Cerebral palsy, Health-related quality of life, Bangladesh, Cerebral palsy quality of life teens, Disability, Adolescent, Psychometric properties, Validation

## Abstract

**Background:**

Quality of life (QoL) and health-related quality of life (HRQoL) measurement in low and middle-income countries of people with cerebral palsy (CP), the major cause of childhood physical disability, is essential to assess the impact of interventions and inform policies that best improve people’s lives. The purpose of this study was to cross-culturally translate and psychometrically validate the Cerebral Palsy Quality of Life-Teens (CPQoL-Teens) self- and proxy-report questionnaires for application with adolescents with CP in Bangladesh.

**Method:**

The CPQoL-Teens questionnaires were translated to Bengali using forward and backwards cross-cultural translation protocols. The questionnaires were interviewer administered to adolescents and their primary caregivers, identified through the Bangladesh Cerebral Palsy Register. Feasibility, sensitivity, internal consistency, content, concurrent and construct validity were assessed.

**Results:**

One hundred fifty four adolescents with CP (10 to 18y; mean 15y 1mo SD 1y 8mo; 31.2% female) participated. Feasibility, sensitivity and internal consistency of both self- and proxy-report questionnaires was excellent; nil missing scores except ‘school wellbeing’ which was associated with non-school attendance (48.4 to 74.7%); floor and ceiling effect ≤13.6%; Cronbach’s alpha 0.77 to 0.94. Instrument validity was good; confirmatory factor analysis reflected five of the seven original instrument dimensions. CPQoL-Teens correlated to Kidscreen-27 on most dimensions (*r* = 0.176 to 0.693, *p* < 0.05); minimal difference in known groups was observed by mental health status (*p* < 0.05) although could be accounted for by homogeneity of mental health problems in the sample.

**Conclusion:**

The CPQoL-Teens self- and proxy report questionnaires successfully translated to Bengali and showed excellent feasibility and strong psychometric properties confirming suitability to assess indicators of HRQoL among adolescents with CP in Bangladesh.

## Background

Quality of life (QoL) and specifically health-related quality of life (HRQoL) assessment is becoming a fundamental component of public health surveillance. QoL is broadly defined as a subjective multidimensional concept for assessing a person’s wellbeing across numerous life indicators [1]. HRQoL is a subset of QoL and used for measuring the interaction between health and indicators of wellbeing [2]. HRQoL is often overlooked in low and middle-income countries (LMICs) in part due to the complexities associated with its measurement however, can provide important understanding of the unique factors impacting people’s lives allowing for focus on positives rather than on deficits (i.e. wellbeing rather than poverty) and contributing to sustainable development [3]. Moreover, HRQoL assessment can be used to deliver valid indicators of intervention outcomes (i.e. assess the impact of clinical interventions and treatment on quality of life as well as health service evaluation); provide understanding of burden of disease; and identification of priority areas for allocation of health resources, public health infrastructure development and policy guidance [4]. HRQoL assessment is particularly relevant to groups with long-term or chronic health conditions and/ or disability such as those with cerebral palsy (CP) and those going through major transitions such as adolescents [5–7].

CP is the major cause of childhood physical disability worldwide and refers to a group of disorders affecting a person’s ability to move or their posture caused by damage to the developing brain either during pregnancy or shortly after birth [8]. It can be associated with co-morbidities including intellectual disability, epilepsy, deafness, blindness and chronic pain among others [9]; and is often more common and more severe in LMICs [10, 11]. A recent population-based study from Bangladesh estimated the prevalence of CP to be 3.4 per 1000 children [12]. 68.2% of the children were unable to walk and prevalence of associated impairments was also notably higher than international norms including visual, hearing impairments and epilepsy. Disability infrastructure and health services are sparse in Bangladesh although as a signatory to the United Nations Convention on the Rights of Persons with Disability and an adoptee of the Sustainable Development Goals, Bangladesh has made considerable efforts to respond to the voices of disability advocates and their allies and reduce inequality by focusing development on holistic concepts of wellbeing [13].

One of the most densely populated and under resourced countries in the world, Bangladesh has a large adolescent population constituting approximately one fifth of the total population. Adolescents in this context face unique circumstances; over 67% of adolescent girls are married and more than 50% will give birth before the age of 18 [14, 15]. Adolescents with CP experience additional challenges as they negotiate their physical, emotional, social and sexual development whilst facing stigma related to their impairments (likely to be severe due to late diagnosis and lifelong lack of services and support [12, 13]) and ostracism due to lack of perceived capacity to undertake narrowly defined adult roles such as being parents and income earners [16]. To date, international research has indicated that some adolescents with CP, primarily from high-income countries (HICs), will experience similar wellbeing to their peers without disability [17] although those from LMICs are likely to lag behind their peers without disability and report extremely poor wellbeing [18]. Condition-specific instruments that study the relationship between CP and individual’s subjective sense of wellbeing and that can be administered in low resource settings are necessary to understand and improve the lives of adolescents with CP [19, 20].

Cerebral Palsy Quality of Life Teens Questionnaire (CPQoL-Teens), an extension of the CPQoL-Child questionnaire, is a widely used condition specific instrument for studying the subjective wellbeing of adolescents with CP using a conceptual framework that aligns quality of life with wellbeing [21]. Originally developed in Australia (an HIC) using a grounded theory approach based on interviews with adolescents with CP and their primary caregivers [22], it is one of five known CP specific HRQoL measures although is the only instrument that is inclusive of indicators specific to adolescents with CP such as pain, participation, social isolation, and feelings about functioning [20]. To date, CPQoL-Teens has been translated to six other languages (Italian, Spanish, Cantonese, Mandarin, Japanese, Hebrew) although has not yet been applied in an LMIC. In the present study, we conducted a cross-cultural translation and adaptation of CPQoL-Teens and assessed the psychometric properties of the self- and proxy-report versions among adolescents with CP and their primary caregivers in Bangladesh.

## Method

This study is part of the Bangladesh cerebral palsy health-related quality of life study (Bangladesh CP HRQoL) aimed at assessing the HRQoL of adolescents with CP in Bangladesh using a population-based sample. The present study reports the cross cultural translation and validation of the CPQoL-Teens questionnaire to determine feasibility, sensitivity, internal consistency, content, concurrent and construct validity for use in Bangladesh.

### Participants and study design

Participants were identified through the Bangladesh Cerebral Palsy Register (BCPR), the first population-based register of children and adolescents with CP in an LMIC [23]. BCPR is an ongoing surveillance program that has been operating since January 2015 and covers a defined geographical region, the Shahjadpur sub-district of Sirajganj district in the northern part of Bangladesh. The area includes 296 villages with a total combined population of 561,076 (child population approx. 226,114) and an estimated 70,998 households. Participants of BCPR are identified using Key Informant Methodology described in Khandaker, Smithers-Sheedy [23]. BCPR holds data on socio-demographic characteristics, clinical (including severity, aetiology, associated impairments and risk factors), nutrition, education and rehabilitation status of children and adolescents with CP in Bangladesh. We attempted to contact all adolescents aged 10 to ≤18-years registered with BCPR at the time of this study to invite participation; ten to 18 is considered a normative classification of adolescence in Bangladesh [24]. Where possible we asked adolescents to self-report; we also requested proxy-reported data from their primary caregiver classified as a parent, grandparent, other relative or close adult friend who provided most of their care and support.

Informed consent was obtained from all individual participants included in the study. In cases of illiteracy, written consent was obtained by thumbprint from the primary caregiver. This study has ethical approval from the Bangladesh Medical Research Council (BMRC/NREC/2013–2016/1165) and University of Sydney Human Research Ethics Committee (2016/646). All procedures performed in this study were in accordance with the ethical standards of these institutional and with the 1964 Helsinki declaration and its later amendments or comparable ethical standards.

### Measures

Self- and proxy-reported (via primary caregivers) data were collected for all participants; adolescents were excluded from self-reporting in instances where they appeared unable to understand questions or communicate their answers. The questionnaires were interviewer-administered by trained workers. Each interview lasted approximately 45 min.

#### Cerebral palsy quality of life – teens (CPQoL-Teens)

CPQoL-Teens is a CP condition specific survey-based instrument that measures subjective wellbeing in teenagers with CP using categorised statistical indicators, see Davis, Mackinnon [21] for the psychometric properties of the original Australian version. CPQoL-Teens has both self- and proxy-report options measuring seven dimensions; ‘general wellbeing and participation’ (21 items), ‘communication and physical health’ (16 items), ‘school wellbeing’ (8 items), ‘social wellbeing’ (7 items), ‘access to services’ (9 items, proxy report only), ‘family health’ (4 items, proxy report only) and ‘feelings about functioning’ (5 items). Participants are asked to rate their feelings on a nine-point Likert scale from 1, ‘very unhappy’ to 9, ‘very happy’ by thinking about how they feel, rather than about what they can do. Each question commences with the phrasing “how do you feel about…” or “How do you think your teenager feels about…”.

#### Bengali version Kidscreen-27

The Bengali version Kidscreen-27 was used to measure concurrent validity. Kidscreen-27 instrument is a generic population survey-based questionnaire that measures HRQoL of children and adolescents [25]. The instrument has self- and proxy-report versions to measure participant subjective perception of their wellbeing over the last week in relation to; ‘physical wellbeing’ (5 items), ‘psychological wellbeing’ (7 items), ‘autonomy and parents’ (7 items), ‘peers and social support’ (4 items), and ‘school environment’ (4 items). The Bengali version Kidscreen-27 reported strong psychometric properties for this study including good internal consistency (Cronbach’s alpha self- and proxy-report 0.7 to 0.9) [26].

#### Bengali version strengths and difficulties questionnaire (SDQ)

The Bengali version Strengths and Difficulties Questionnaire (SDQ) was used to measure construct validity. SDQ provides an assessment on mental health including emotional symptoms (5 items), conduct problems (5 items), hyperactivity/ inattention (5 items), peer relationship problems (5 items), and pro-social behaviour (5 items) of children and adolescents. The Bengali version SDQ has previously been validated for use in Bangladesh [27].

#### Bangladesh cerebral palsy register (BCPR)

Demographic and disability related information were extracted from the BCPR database including age, sex, height, weight, gross motor function classification system (GMFCS) level, associated impairments, level of education and monthly family income [12].

### Cross-cultural translation and adaptation of CPQoL-teens

Translation and adaptation of the self- and proxy-report questionnaires followed the CPQoL-Teens forwards and back translation protocol [28] with necessary socio-cultural adaptations to attain language, operational and scale equivalence [29, 30]. The self- and proxy-report English versions were independently translated by two researchers fluent in both languages but for whom Bengali was their day-to-day language. The translators were given instruction to use natural and acceptable language for the broadest audience and to be simple, clear and concise in their formulations, as well as to focus on conceptual equivalence rather than literal word-for-word translation. The two translations were compared and assessed for conceptual equivalence, comprehensibility and clarity of speech relative to the original English questionnaires. A reconciled version of each questionnaire was produced as a derivative of the two translations.

The reconciled forward translation was back translated by a researcher fluent in both languages but for whom English was their day-to-day language. The same instructions for translation were given to the English translator. The back translation was then compared item-by-item with the original English questionnaires to develop the final forward translation document. Conceptual discrepancies between the translations were resolved, involving a researcher experienced in CP, who analysed each item and chose the best translation, or suggested another translation if necessary. Pre-testing of the final self- and proxy-report questionnaires were undertaken with eight adolescents and primary caregivers to confirm the translations. Questions were discussed and reformulated by the researchers if understood by < 90% of participants, then tested with another group of eight adolescents with CP and primary caregivers. Acceptability of the instrument administration method, timeframe required for administration and use of the Likert scale were also assessed. This process was repeated until all items were understood by > 90% of participants. The translated questionnaires are available on request from CPQoL.

### Statistical analysis

Data were checked for accuracy and records where age was invalid were excluded from analysis. CPQoL-Teens scores were converted to values between 0 and 100, selected scores inverted so that higher scores indicated better HRQoL, and summary scores calculated by averaging the items with each dimension [28]. Dimension (average) scores were used for all subsequent analysis.

Feasibility was assessed as the proportion of missing values and was analysed by case-wise deletion. Instrument sensitivity was assessed using floor and ceiling effects, defined as the proportion of participants reporting the lowest and highest scores for each instrument dimension. Floor or ceiling effects > 15% were considered as high indicating that the instrument is not sensitive in the target population [25]. Internal consistency was calculated using Cronbach’s α. This coefficient has a value from 0 to 1; a value ≥0.7 was considered to indicate high reliability of the instrument for use in group comparison and a value ≥0.9 indicates high reliability for individual patient analysis [31, 32].

Content validity was assessed using confirmatory factor analysis (CFA) to confirm if the underlying dimensions of the translated questionnaire matched the original. Model fit was considered acceptable if the chi-square statistic was *p* > 0.05 and root mean squared error of approximation (RMSEA) was ≤0.08, comparative fit index (CFI) was ≥0.90 and Tucker-Lewis Index (TLI) was ≥0.90 [33]. Exploratory factor analysis (EFA) was undertaken if CFA was determined a poor model fit [34]. We conducted principal component analysis with Varimax rotation; factors were disregarded according to visual inspection of Scree plot and if eigenvalue was < 1.0. Forced extraction was conducted to achieve most interpretable solution [34].

Concurrent validity was assessed by determining the degree of correlation between related dimensions of CPQoL-Teens and Kidscreen-27. Coefficients exceeding r = 0.7 were considered satisfactory [35]. Construct validity was determined using the known group’s method [2]. We assessed differences according presence of ‘unlikely’, ‘possible’ and ‘probable’ mental health problems using SDQ [25]. Magnitude of difference was determined by effect size as small (≤0.49), medium (0.50 to 0.79), and large (≥0.80) (Cohen 1988). Concordance between self-report and proxy-report was assessed with intra class correlation (ICC) and comparison of group means. ICC < 0.4 was considered to indicate poor to fair agreement, 0.50 to 0.69 moderate agreement, 0.70 to 0.79 good agreement, > 0.80 excellent agreement [36]. All statistical analysis was conducted using SPSS version 24 (IBM Corporation, Chicago, Illinois, USA). A *p* value of < 0.05 was considered significant.

## Results

### Participant characteristics

One hundred ninety two adolescents with CP were registered in BCPR at the time of this study, of which 154 (mean age 15y 1mo, SD 1y 8mo, female *n* = 48, 31.2%) were enrolled to participate (participation rate 80.2%). Reasons for non-participation included being unwilling to participate (*n* = 11, 5.7%); no longer living in the surveillance area (*n* = 7, 3.6%); not able to be retraced (*n* = 17, 8.9%); and having deceased (*n* = 3, 1.6%). Of the 154 adolescents with CP, 64 (41.56%) provided self-reported data. Remainder were excluded from self-reporting due to severe communication and/ or cognitive impairment. Proxy-reported data was provided for all adolescents by primary caregivers; mothers (*n* = 118, 76.62%), fathers (*n* = 21, 13.64%) and other primary caregivers (*n* = 15, 9.74%), mean caregiver age 39y 9mo (SD 9y 9mo). Majority of adolescents with CP were underweight (BMI < 18.5 *n* = 107, 69.48%) and did not attend school (*n* = 115, 74.68%). Participant GMFCS was Level I *n* = 36 (23.38%), Level II *n* = 23 (14.94%), level III *n* = 33(21.43%), level IV *n* = 20 (12.99%), level V *n* = 41 (26.62%). Median monthly family income was BDT 6000 (USD equiv. $73).

### Feasibility

Missing values, shown in Table 1, were nil for all dimensions except ‘school wellbeing’ (missing self-report 48.4%; proxy-report 74.7%). Missing scores corresponded exactly to rates of non-school attendance.

**Table 1:** Feasibility (missing scores), sensitivity (floor and ceiling effect) and internal consistency (Cronbach’s α), of the Bengali version CPQoL-Teens

Instrument dimension	Items	Adolescent with CP				
		*n*	Missing scores *n*(%)	Floor effect *n*(%)	Ceiling effect *n*(%)	Cronbach’s α
Self-report						
General wellbeing and participation	21	*64*	0(0.0)	0(0.0)	0(0.0)	0.92
Communication and physical health	16	*64*	0(0.0)	0(0.0)	0(0.0)	0.85
School wellbeing	8	*64*	31(48.4)	0(0.0)	3(4.7)	0.89
Social wellbeing	7	*64*	0(0.0)	0(0.0)	2(3.1)	0.77
Feelings about functioning	5	*64*	0(0.0)	1(1.6)	2(3.1)	0.87
Proxy-report						
General wellbeing and participation	21	*154*	0(0.0)	0(0.0)	0(0.0)	0.94
Communication and physical health	16	*154*	0(0.0)	2(1.3)	0(0.0)	0.88
School wellbeing	8	*154*	115(74.7)	0(0.0)	1(0.7)	0.91
Social wellbeing	7	*154*	0(0.0)	2(1.3)	1(0.7)	0.82
Access to services	9	*154*	0(0.0)	1(0.7)	2(1.3)	0.88
Family health	4	*154*	0(0.0)	5(3.3)	2(1.3)	0.79
Feelings about functioning	5	*154*	0(0.0)	21(13.6)	2(1.3)	0.88

### Sensitivity

Floor and ceiling effects, Table 1, were observed as nil or weak on most dimensions for both self- and proxy-report questionnaires (0 to 4.7%). Moderate ceiling effect was observed in proxy-report ‘feelings about functioning’ (13.6%). No effects > 15% were observed. Sub-group analysis by BMI, school attendance and monthly family income revealed no significant floor or ceiling effects confirming good sensitivity.

### Internal consistency

Internal consistency, Table 1, was excellent; Cronbach’s α self-report 0.77 to 0.92 and proxy-report 0.79 to 0.94.

### Validity

#### Content validity

CFA on the Bengali proxy-report questionnaire showed the original seven factor model was a poor fit (Chi-square = 5593.9, df = 2326, *p* < 0.001, RMSEA = 0.065 (95% CI 0.063 to 0.068); CFI = 0.612; TLI = 0.586). EFA was then conducted as sampling adequacy was acceptable (Bartlett = < 0.001, KMO = 0.858). This resulted in a 15 factor solution with eigenvalues greater than one, explaining 75.7% of the variance. Forced factor extraction resulted in a final five-factor solution that explained 54.8% of the variance. This solution was most interpretable and corresponded, within one to three factors, with the original CPQoL-Teens dimensions, see Table 2. Final factor loadings for all factors ranged from 0.26 to 0.84. CFA was not conducted for the Bengali CPQoL-Teens self-report questionnaire due to insufficient sample size (*n* = 64).

**Table 2:** Content Validity. CPQoL-Teens proxy-report item factor loadings

	Factor 1	Factor 2	Factor 3	Factor 4	Factor 5
General wellbeing and participation					
1. their ability to participate in leisure and recreational activities?	**0.77**				
2. their ability to participate in the community?	**0.77**				
3. their ability to participate in social events outside of school?	**0.76**				
4. having a go and trying new things?	**0.74**			−0.31	
5. succeeding in things they want to be good at?	**0.72**				
6. their positive attitude?	**0.71**				
7. their ability to participate in sporting activities?	**0.70**				
8. doing things they want to do?	**0.69**				
9. their ability to get around in their neighbourhood?	**0.69**				
10. themselves?	**0.69**				
11. they way their are accepted by other teenagers outside of school?	**0.68**				
12. their ability to get from place to place?	**0.66**		0.31		
13. hanging out with friends?	**0.65**				
14. they way they get along with other teenagers outside of school (not school friends)?	**0.64**				
15. their opportunities in life?	**0.64**				
16. their future?	**0.58**				
17. quality of life?	**0.56**			0.43	0.33
18. life as a whole?	0.52			**0.52**	0.31
19. the way they get around?	**0.52**			0.32	
20. life in general?	**0.48**			0.45	0.33
21. hanging out on their own?	**0.48**			0.38	
Communication and physical health					
22. their ability to communicate with people they know well?	**0.75**				
23. being able to do things by themselves without relying on others?	**0.72**				
24. the way other people communicate with them?	**0.72**				
25. the way they get along with adults?	**0.67**			−0.34	
26. what they have achieved in their life?	**0.66**				
27. their physical health?	**0.62**			0.33	
28. the way they are accepted by adults?	**0.62**				
29. their plans for the future?	**0.59**				
30. changes happening to their body to do with puberty?	**0.58**				
31. their overall health?	**0.57**			0.34	
32. the way they are accepted by people in general?	**0.56**	0.34			
33. their ability to communicate with people they do not know well?	**0.56**				
34. how they sleep?	**0.47**				
35. what may happen to them later in life?	**0.44**				
36. the way their communicate with people using technology (SMS, internet)?	**0.41**				
37. their ability to keep up academically?		**0.59**			
School wellbeing					
38. the way they are treated the same as everyone as at school?		**0.84**			
39. their ability to keep up physically?		**0.77**			
40. their ability to participate at school?		**0.77**			
41. the way they get along with their teachers at school?		**0.75**			
42. the way they get along with other teenagers at school?		**0.74**			
43. the way they are included by other students at school?		**0.74**			
44. the way they are accepted by other students at school?		**0.72**			
45. the way they are accepted by staff and teachers at school?		**0.66**			
Social wellbeing					
46. the way they get along with you (parents)?	**0.70**				
47. the way they get along with people generally?	**0.62**				
48. how happy they are?	**0.57**			0.34	0.30
49. going out on trips with the family?	**0.57**		0.38		
50. the way they get along with their brothers and sisters?	**0.55**				
51. the support they get from their family?	**0.43**			−0.34	0.31
52. the way they are accepted by their family?	**0.42**		0.33		
Access to services					
53. your teenagers access to treatment?	0.53	0.31	**−0.55**		
54. your teenagers access to community services and facilities?	**0.49**	0.34			−0.34
55. your teenagers access to speech therapy?	0.45		**−0.69**		
56. ability to get advice from a paediatrician?	0.45	0.35	**−0.63**		
57. your teenagers access to specialised medical or surgical care?	0.43	0.41	**−0.65**		
58. your teenagers access to physiotherapy?	0.43	0.34	**−0.69**		
59. your teenagers access to occupational therapy?	0.42	0.34	**−0.69**		
60. your teenagers access to extra help with learning at school?		**0.70**			
61. How much pain does your teenager have?			**0.26**		
Family health					
62. How happy are you?	0.41				**0.45**
63. Your family’s financial situation?	**0.41**				0.32
64. Your work situation?	0.31		−0.36		**0.57**
65. Your physical health?			−0.31		**0.54**
Feelings about functioning					
66. their ability to dress him/herself?	**0.63**				
67. their ability to eat or drink independently?	**0.60**				
68. the way they use their arms and hands?	**0.59**				
69. their ability to use the toilet by themself?	**0.58**			0.33	
70. the way they use their legs?	0.44			**0.46**	−0.31
Eigenvalue	**20.47**	**7.06**	**4.52**	**3.53**	**2.74**
Percent variance	**29.25%**	**10.08%**	**6.46%**	**5.05%**	**3.91%**

Extraction method: Principal component analysis; Rotation method: Varimax with Kaiser Normalization; Primary loadings are in bold

#### Concurrent validity

Concurrent validity between CPQoL-Teens and Kidscreen-27 was good, as shown in Table 3. Significant moderate to strong correlation was observed in self-reported versions of the questionnaires on all dimensions (*r* = 0.257 to 0.693, *p* < 0.05) with two exceptions (CPQoL-Teens ‘social wellbeing’ to Kidscreen-27 ‘peers & social’ and CPQoL-Teens ‘feelings about functioning’ to Kidscreen-27 ‘school wellbeing’ *p* > 0.05). Significant weak to strong correlations were observed in the proxy report version of the two questionnaires on all dimensions (*r* = 0.173 to 0.648, *p* < 0.05) with ten exceptions (CPQoL-Teens ‘school wellbeing’ to Kidscreen-27 ‘total score’, ‘psychological wellbeing’, ‘autonomy and parents’, and ‘peers and social’; CPQoL-Teens ‘social well-being’ to Kidscreen-27 ‘school wellbeing’; CPQoL-Teens ‘access to services’ to Kidscreen-27 ‘psychological wellbeing’, ‘autonomy and parents’ and ‘school wellbeing’; and CPQoL-Teens ‘family health’ to ‘autonomy and parents’ and ‘school wellbeing’, *p* > 0.05).

**Table 3:** Concurrent validity. Spearman’s correlation between CPQoL-Teens and Kidscreen-27 self- and proxy-reports

CPQoL-Teens	Kidscreen-27					
	Total score	Physical wellbeing	Psych wellbeing	Autonomy parents	Peers social	School wellbeing
Self-report						
General wellbeing and participation	.495**	.556**	.532**	.390**	.346**	.460**
Communication and physical health	.505**	.553**	.459**	.465**	.370**	.659**
School wellbeing^a^	.693**	.663**	.458**	.545**	.490**	.563**
Social wellbeing	.257*	.374**	.378**	.367**	.171	.377*
Feelings about functioning	.485**	.537**	.598**	.313*	.540**	.319
Proxy-report						
General wellbeing and participation	.648**	.555**	.568**	.474**	.544**	.352*
Communication and physical health	.603**	.476**	.513**	.439**	.555**	.425**
School wellbeing^b^	.225	.365*	.062	.043	.216	.327*
Social wellbeing	.479**	.389**	.477**	.366**	.357**	.054
Feelings about functioning	.529**	.598**	.447**	.295**	.467**	.391*
Access to services	.226**	.171*	.137	.099	.173*	.106
Family health	.269**	.257**	.391**	.120	.243**	.082

** correlation is significant at 0.01 level; * correlation is significant at the 0.05 level

^a^ *n* = 33; ^b^ *n* = 39

#### Construct validity

The differences between ‘known groups’ according to SDQ mental health status (‘unlikely’, ‘possible’ or ‘probable’ mental health problem) are shown in Table 4. Group difference according to SDQ mental health status was observed in ‘communication and physical health’ and ‘feelings about functioning’ (mean difference 9.7 (0.6 to 18.9) to 17.8 (0.7 to 35.0), ES < 0.4, *p* < 0.05) although was insignificant for all other dimensions (*p* > 0.05). No difference was observed when analysed by socioeconomic status measured as monthly family income (*p* > 0.05).

**Table 4:** Known group differences. Mean difference in CPQoL-Teens proxy-reported scores according to mental health status (SDQ)

Instrument dimension	SDQ ‘unlikely’ to ‘possible’ mean difference (95% CI)	SDQ ‘unlikely’ to ‘probable’ mean difference (95% CI)	SDQ ‘possible’ to ‘probable’ mean difference (95% CI)	Effect size (ES)
General wellbeing and participation	11.3 (−2.1 to 24.7)	9.1 (−1.9 to 20.2)	2.2 (−8.3 to 12.6)	0.03
Communication and physical health	13.4* (2.2 to 24.5)	9.7* (0.6 to 18.9)	3.6 (−5.0 to 12.3)	0.06
School wellbeing ^a^	8.8 (−16.2 to 33.7)	1.1 (− 16.0 to 18.3)	7.6 (−14.8 to 30.0)	0.02
Social wellbeing	11.7 (−0.7 to 24.1)	8.1 (−2.2 to 18.3)	3.7 (−6.0 to 13.3)	0.03
Access to services	2.1 (−13.8 to 18.1)	4.5 (−8.7 to 17.6)	6.6 (−5.8 to 19.0)	0.01
Family health	8.6 (−6.1 to 23.2)	9.2 (−2.9 to 21.3)	0.6 (−10.8 to 12.0)	0.02
Feelings about functioning	17.8* (0.7 to 35.0)	7.6 (−6.6 to 21.7)	10.3 (−3.1 to 23.6)	0.04

* significant at the 0.05 level

^a^ *n* = 39

### Concordance between self- and proxy-report

Concordance between self- and proxy-report, Table 5, was moderate to excellent for all dimensions (ICC 0.5 to 0.9). In all instances proxies estimated poorer HRQoL than adolescents themselves (mean difference 6.3 to 7.7 *p* < 0.05).

**Table 5:** Concordance between self- and proxy-reports. ICC and mean difference between self- and proxy-reported CPQoL-Teens (*n* = 64)

Instrument Dimension	ICC (95% CI)	Mean difference (95% CI)	*p*-value
General wellbeing and participation	0.8 (0.7 to 0.9)	6.5 (3.3 to 9.6)	**< 0.0001** ^b^
Communication and physical health	0.8 (0.6 to 0.9)	6.7 (3.6 to 9.7)	**< 0.0001** ^b^
School wellbeing ^a^	0.9 (0.7 to 0.9)	7.7 (4.2 to 11.3)	**< 0.0001** ^b^
Social wellbeing	0.5 (0.2 to 0.7)	7.0 (3.1 to 10.9)	**0.001** ^c^
Feelings about functioning	0.7 (0.4 to 0.8)	6.3 (0.2 to 12.4)	**0.021** ^c^

^a^ *n* = 33; ^b^ paired samples *t*-test; ^c^ Wilcoxon signed rank test

## Discussion

To the best of our knowledge this is the first validation study of CPQoL-Teens in an LMIC using a population-based sample of adolescents with CP. Our study demonstrated that the Bengali version CPQoL-Teens self- and proxy-report questionnaires have overall good psychometric properties and are reliable and valid measures to assess indicators of HRQoL among adolescents with CP in Bangladesh. We followed a rigorous cross-cultural translation and adaptation procedure; we used multistage forward and back translation with pilot testing to attain appropriate language and socio-cultural adaptations and adjusted instrument administration (questionnaires were interviewer administered) to account for low levels of literacy within our target population [12]. Acceptability of instrument administration method, timeframe and use of Likert scale measurement were confirmed during pilot testing.

The Bengali version CPQoL-Teens self- and proxy-report questionnaires produced psychometric properties comparable to the original Australian versions, with minor variations [21]. Missing scores were < 5% in the Australian version (for sub-dimensions included in the final questionnaire). Our study reported nil missing scores except in ‘school wellbeing’ (due to non-school attendance in the sample rather than an administration issue, discussed shortly). The Australian versions reported no floor effects; ceiling effects were 1–7%. The Bengali versions study reported weak floor and ceiling effects (range 0 to 4.7%) with the exception of a moderate floor effect in the proxy version ‘feelings about functioning’. This dimension should be monitored in future applications of CPQoL-Teens although is a likely reflection of the lifelong lack of therapy and treatment options and subsequent severity of impairment among children with CP in Bangladesh [12]. Regarding internal consistency, Cronbach’s α in the Australian CPQoL-Teens was 0.78 to 0.96 self-report and 0.81 to 0.96 proxy-report. Internal consistency was similarly excellent in the Bengali versions exceeding scale cut offs for group comparison (Cronbach’s α < 0.70), although further testing is recommended before use in individual patient analysis as most dimensions were Cronbach’s α < 0.90.

Adolescents with CP in our sample reported high rates of mental health problems (19.5% ‘possible’ and 63.6% ‘probable’) although this is similar to prevalence of mental health problems amongst children and adolescents with CP reported elsewhere [37]; and all participants lived in a rural area of Bangladesh and had similar levels of monthly family income. The Bengali version CPQoL-Teens did not discriminate well between groups on the basis of these characteristics however this is likely due to homogeneity in our sample. Previous studies that have used these characteristics to test known group difference have conducted assessment with more sensitive measures including the ‘Family Affluence Scale’ (FAS) however use of FAS was not feasible in our low-resource setting [38, 39]. Administration of CPQoL-Teens amongst adolescents with CP in other geographic regions of Bangladesh is recommended to further testing to known group difference.

We identified a high proportion of missing data in ‘school wellbeing’ associated with non-school attendance, which is common for adolescents with disability in Bangladesh [40]. These findings indicated that the Bengali translated CPQoL-Teens may address indicators of HRQoL that are not universally applicable for adolescents with CP in Bangladesh. Supplementary dimensions may be required for the Bangladeshi context, for example, to capture QoL data about non-school attendance for Bangladeshi adolescents with CP. This is an area for future research.

Concurrent validity examined agreement between sub-dimensions in the Bengali CPQoL-Teens and Bengali Kidscreen-27. Moderate to strong correlation was observed between most but not all sub-dimensions. Lack of correlation is suggested to be the result of conceptual differences in the instruments. Kidscreen-27 focuses on activities (e.g. “Thinking about the last week, have you …) whereas CPQoL-Teens focus on feelings (e.g. “How do you feel about…”).

Self-report is the gold standard of QoL assessment although exclusive use of proxy-reported data is common in HRQoL studies in LMICs [18]. We anticipated due to high prevalence of severe communication and/ or cognitive impairment that numerous adolescents would not be able to self-report however we ensured that in all instances data were collected directly from the adolescent as well as from their primary caregiver; this was possible in 41.6% of cases. To understand the agreement between self- and proxy-report we conducted case-wise comparison. Dimensions with observable components, for example, ‘general wellbeing and participation’ showed stronger agreement and dimensions with non-observable emotional components, for example ‘social wellbeing’ showed weaker agreement. Further discussion on the pattern of self- vs proxy-reported scores is provided in Power, Muhit [41]. Our findings of discordance were consistent with other research [42, 43] and provide strong argument for research design to include methods that enable collection of self-reported data.

Measure of HRQoL is widely adopted in HICs and majority of HRQoL instruments originate from these settings [44], as was the case with CPQoL-Teens. Cross-cultural translation and adaptation of HRQoL instruments can be complex, lacking local context, however are useful from a public health perspective to identify international trends and enable collaboration [3, 29]. Moreover, argument can be made that although context will vary between HICs and LMICs, people with disability share similar social positions (i.e. in regards to marginalisation and oppression) providing a point of unity when conducting cross-cultural HRQoL assessment [7, 45]. Determining how HRQoL is conceptualised amongst our target population and providing commentary on the theoretical developments of ‘wellness’ was beyond our scope; although analysis of instrument dimensions confirmed that CPQoL-Teens reflected internationally defined multidimensional theoretical constructs of HRQoL [2, 3]. Moreover, assessment of content validity on the proxy-report questionnaire reflected five of the seven dimensions of the originals CPQoL-Teens although further testing with larger samples is required to confirm the factor structure.

### Limitations

A limitation of this study is that the questionnaires were originally developed in high-income (western) countries for use with children/ adolescents of defined ages, which differed to our age classification of adolescence in Bangladesh. Specifically CPQoL-Teens was originally developed for use with 13 to 18 year olds, SDQ for use with 3 to 16 year olds, and Kidscreen-27 for use with 8 to 18 year olds. This study considered adolescents in Bangladesh as 10 to 18 years, noted previously; meaning the instruments were applied outside of the age range for which they were originally developed and validated.

A further limitation of this study is that due to resource constraints we do not measure test-retest reliability or structural validity however we provide a thorough analysis of feasibility, sensitivity, internal consistency, content, concurrent and construct validity. Further testing of both questionnaires with samples from different geographical regions of Bangladesh would complement our findings.

## Conclusion

CPQoL-Teens self- and proxy-report questionnaires underwent rigorous procedure for cross-cultural translation and adaptation and reported strong psychometric properties indicating suitability for use assessing the HRQoL of adolescents with CP in an LMIC, Bangladesh. The Bengali version CPQoL-Teens questionnaires were sensitive for adolescents with CP and captured information specific to this cohort, although relevance of ‘school wellbeing’ requires further investigation due to high levels of non-school attendance within our surveillance area. The questionnaires indicated good internal consistency for use in group comparison. Psychometric validation of CPQoL-Teens for use in Bangladesh is an important development to enable holistic assessment of adolescent’s wellbeing in an LMICs and guide disability infrastructure development and policies.

## Data Availability

The datasets used and/or analysed during the current study are available from the corresponding author on reasonable request.

## Abbreviations

CP: Cerebral palsy
CPQoL-Teens: Cerebral Palsy Quality of Life Teens Questionnaire
ES: Effect size
GMFCS: Gross motor function classification system
HICs: High-income countries
HRQoL: Health-related quality of life
LMICs: Low and middle-income countries
SD: Standard deviation
